# Development of Cadmium Multiple-Signal Biosensing and Bioadsorption Systems Based on Artificial *Cad* Operons

**DOI:** 10.3389/fbioe.2021.585617

**Published:** 2021-02-10

**Authors:** Yan Guo, Chang-ye Hui, Nai-xing Zhang, Lisa Liu, Hui Li, Hong-ju Zheng

**Affiliations:** ^1^National Key Clinical Specialty of Occupational Diseases, Shenzhen Prevention and Treatment Center for Occupational Diseases, Shenzhen, China; ^2^Department of Pathology and Toxicology, Shenzhen Prevention and Treatment Center for Occupational Diseases, Shenzhen, China; ^3^Institute of Translational Medicine, Shenzhen Second People's Hospital, Shenzhen, China

**Keywords:** *cad* operon, *E coli*, whole-cell biosensor, cadmium detection, cadmium adsorption

## Abstract

The development of genetic engineering, especially synthetic biology, greatly contributes to the development of novel metal biosensors. The *cad* operon encoding cadmium resistance was previously characterized from *Pseudomonas putida*. In this study, single-, dual-, and triple-signal output Cd(II) biosensors were successfully developed using artificial translationally coupled *cad* operons. Sensitivity, selectivity, and response toward Cd(II) and Hg(II), of three biosensors were all determined. Reporter signals of three biosensors all increased within the range 0.1–3.125 μM Cd(II). Three biosensors responded strongly to Cd(II), and weakly to Hg(II). However, the detection ranges of Cd(II) and Hg(II) do not overlap in all three biosensors. Next, novel Cd(II) biosensing coupled with bioadsorptive artificial *cad* operons were assembled for the first time. Cd(II)-induced fluorescence emission, enzymatic indication, and Cd(II) binding protein surface display can be achieved simultaneously. This study provides an example of one way to realize multiple signal outputs and bioadsorption based on the redesigned heavy metal resistance operons, which may be a potential strategy for biodetection and removal of toxic metal in the environment, facilitating the study of the mechanism and dynamics of bioremediation.

## Introduction

Widespread distribution of heavy metals mostly results from human industrial and agricultural activities, and the presence of toxic heavy metals exerts adverse effects on the environment as well as on human health (WHO, 2010). Some toxic heavy metals such as lead, mercury, and cadmium have been associated with human health concerns at the microgram level in food and drinking water (Rehman et al., 2018). Environmental cadmium is easily absorbed by plants, and accumulated through the food chain. Cadmium pollution has always been a priority in food contaminations (Mie et al., 2017; Genthe et al., 2018).

The advantages of traditional instrumental analysis for cadmium such as atomic absorption spectroscopy and inductively coupled plasma mass spectrometry are accuracy and sensitivity (Wanekaya et al., 2008). However, these analytic methods are costly and time-consuming. More importantly, they cannot provide useful information on the bioavailability of cadmium, which is closely related to its toxic effects on living organisms. Whole-cell biosensors have the potential to complement existing chemical and physical methods for monitoring the bioavailability and biological effects of heavy metals (Frazzoli et al., 2007; Cerminati et al., 2015; Cui et al., 2018).

Generally, a whole-cell biosensor is a genetically engineered cell that generates a reporter signal in response to a target metal ion. It is commonly comprised of a sensing element for recognizing the specific metal ion, and a reporter element for signal output (Gupta et al., 2019). The development of synthetic biology has greatly facilitated the design of biosensors. ZntA, CadC, and CadR have been identified as heavy metal-responsive transcription factors from *zntA* operon of *Escherichia coli* (Brocklehurst et al., 1999), *cadCA* operon of *Staphylococcus aureus* (Endo and Silver, 1995), and *cad* operon of *Pseudomonas putida* (Lee et al., 2001; Brocklehurst et al., 2003), respectively. The resistance mechanisms are involved in the Cd(II) inducing expression of the Cd(II) export genes. Several Cd(II) biosensors using these heavy metal-responsive transcription factors as the sensing elements were successfully developed. Previously reported whole-cell Cd(II) biosensors rely mainly on a single-signal output, such as fluorescent proteins (Tao et al., 2013; Kim et al., 2016; Yoon et al., 2016a; Bereza-Malcolm et al., 2017), reporter enzymes (Tauriainen et al., 1998), and pigment accumulation (Joe et al., 2012). Any kind of detection method has its own shortcomings, so multiple-signal outputs can provide more information and more available detection ways. Fluorescent proteins are the most commonly used reporters and can be directly detected without extra substrates. Many fluorescent protein variants which have emerged for reporter constructs are currently available (Shaner et al., 2004). Fluorescent proteins of different colors also exhibit their advantages. Green fluorescent protein (GFP) has an intrinsic ability to produce fluorescence in the absence of cofactors, so it is the most widely used fluorescent reporter for metal biosensors (Tsien, 1998). A series of spectral variants of GFP have been generated by mutagenesis. Among them, the emitted fluorescence intensity of eGFP is the highest, and so the low level expression of eGFP can also be fluorescently detectable. This is beneficial as it reduces the detection limit of biosensors (Nadarajan et al., 2014; Kim et al., 2016; Yoon et al., 2016b). Due to the bright color of red fluorescent protein (RFP), RFP variants are usually used as reporters for metal biosensing, and the accumulation of RFP can be easily distinguished from the background by the naked eye (Wei et al., 2014; Hui et al., 2018c; Yan et al., 2018). The greatest advantage of an enzymatic reporter is its high sensitivity. Whole-cell Hg(II) biosensors were previously constructed by fusing the mercury inducible promoter and its regulatory factor MerR with reporter genes *LuxCDABE, lacZYA*, or *gfp*. The detection limit of enzymatic reporter β-galactosidase is similar to that of luciferase, but 100 times less than that of fluorescent reporter GFP (Hansen and Sorensen, 2000).

In this study, based on a natural *cad* operon derived from *Pseudomonas putida* 06909 (Lee et al., 2001), a series of artificial *cad* operons were designed, constructed, and validated for multiple-signal biosensing and adsorption of cadmium. The *cadR* and its divergent promoter was fused with a monocistronic mCherry, a dicistronic mCherry-eGFP, or a tricistronic mCherry-eGFP-lacZα genetic element to generate a single-signal output, a dual-signal output, or a triple-signal output Cd(II) biosensor construct, respectively. To maximize collaborative reporting, reporter genes are arranged in the polycistronic construct according to their reporting abilities. It allows them to be co-transcribed into a polycistronic mRNA strand, followed by coupled production of both fluorescent and enzymatic signals. The three different biosensors presented above were finally tested through sensitivity and selectivity analyses. Co-expression of multiple reporters was achieved in polycistronic biosensor constructs, and the upstream reporter was just slightly decreased by the introduction of the downstream reporter. After the mCherry reporter element was substituted by Cd(II) binding protein surface display genetic element, versatile Cd(II) biosensing-adsorptive constructs were then assembled. Fluorescent visualization, enzymatic indication, and Cd(II) removal can be achieved in the meantime using this novel artificial *cad* operon construct. To the best of our knowledge, this is the first report about whole-cell multiple-signal biosensing and adsorption of Cd(II). This study provides an example of one way to realize fluorescent signal output, enzymatic signal output, and even bioadsorption simultaneously based on the artificial MerR family operons upon target heavy metal exposure.

## Materials and Methods

### Bacterial Strains, Plasmids, and Agents

The bacterial strain and plasmids involved in this study are listed in Table 1. *E. coli* TOP10 was used as the host, and cultured in M9 medium (0.68% Na_2_HPO_4_, 0.3% KH_2_PO_4_, 0.05% NaCl, 0.1% NH_4_Cl, 0.01% MgSO_4_, and 0.001% CaCl_2_) supplemented with glucose at 0.4%, IPTG at 0.1 mM, and ampicillin at 50 μg/mL. Stock solutions of CaCl_2_, MgCl_2_, FeSO_4_, MnSO_4_, NiSO_4_, CuSO_4_, ZnSO_4_, CdCl_2_, Pb(NO_3_)_2_, and HgCl_2_ were freshly prepared with analytical grade chemicals and distilled water. SP sepharose fast flow was obtained from GE Healthcare (USA). Isopropyl-β-D-thiogalactopyranoside (IPTG) and o-nitrophenyl-β-D-galactopyranoside (ONPG) were obtained from Sangon Biotech (Shanghai, China). All oligonucleotide primers and DNA fragments were synthesized by Sangon Biotech (Shanghai, China).

**Table 1:** Bacterial strains and plasmids used in this study.

**Strains/plasmids**	**Genotypes or description**	**Source**
*E. coli* Top10	F^−^, Φ80*lac*ZΔM15, Δ*lac*X74, *rec*A1	Invitrogen
pET-21a	Amp^R^, T7 promoter, lac operator	Novagen
pT-RFP	T vector carrying *mcherry*	Hui et al., 2018c
pT-GFP	T vector carrying *egfp*	Hui et al., 2018c
pPlac-lacZα	Amp^R^, *ori pMB1*, pBR322 derivative with lacZα peptide expressing under *lac* promoter	Guo et al., 2019
pT-Pcad	T vector carrying *cadR* and Pcad divergent promoter region	This study
pT-CdBD	T vector carrying *lpp-ompA-CdBD*	This study
pPcad	pET-21a derivative containing *cadR*, Pcad divergent promoter region	This study
pPcad-RFP	pPcad derivative carrying promoterless *mcherry* cloned into *Nde*I and *Hin*dIII sites	This study
pPcad-RFP-GFP	pPcad derivative, an artificial two-cistron *cad* operon with a translationally coupled *mcherry*-*egfp* cassette	This study
pPcad-RFP-GFP-lacZα	pPcad derivative, an artificial three-cistron *cad* operon with a translationally coupled *mcherry*-*egfp*-*lacZα* cassette	This study
pPcad-LOA	pPcad derivative carrying promoterless *lpp-ompA* cloned into *Nde*I and *Hin*dIII sites	This study
pPcad-CdBD	pPcad derivative carrying promoterless *lpp-ompA-CdBD* cloned into *Nde*I and *Hin*dIII sites	This study
pPcad-CdBD-GFP	pPcad derivative, an artificial two-cistron *cad* operon with a translationally coupled *lpp-ompA-CdBD*-*egfp* cassette	This study
pPcad-CdBD-GFP-lacZα	pPcad derivative, an artificial three-cistron *cad* operon with a translationally coupled *lpp-ompA-CdBD*-*egfp*-*lacZα* cassette	This study

*The cloning/expression regions of recombinant plasmids used in this study are shown in Supplementary Figure 1*.

### Construction of the Plasmids for Biosensing and Adsorption of Cadmium

**Figure 1:**
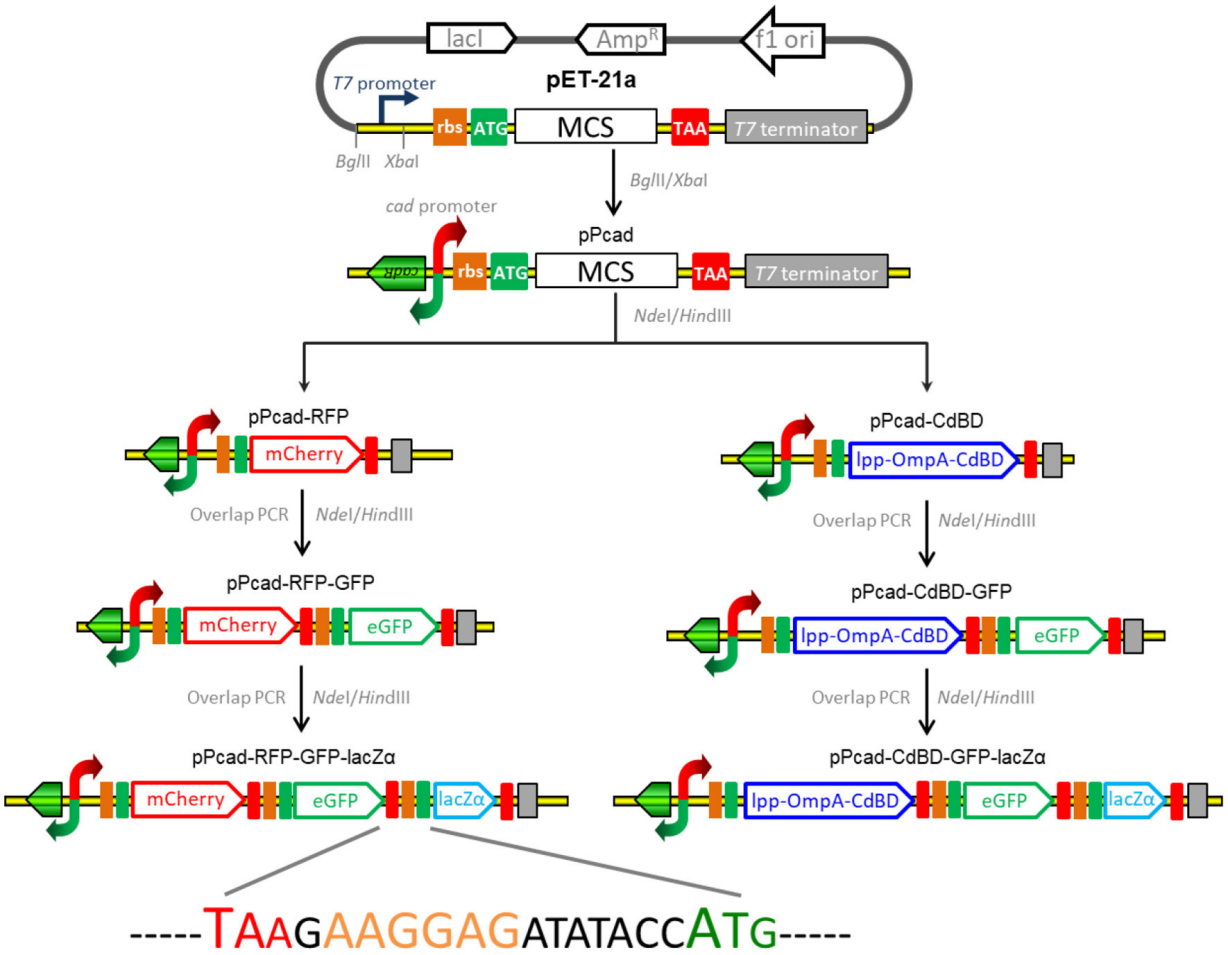
Assembly of artificial *cad* operons for multiple-signal biosensing and adsorption of Cd(II). Three reporters were placed under the control of a *cad* promoter separately or in combination (left panel). The outer membrane anchoring chimera protein and reporters were placed under the control of the *cad* promoter separately or in combination as shown above (right panel). The DNA sequence containing the stop codon of the upstream gene, an extra RBS, and the start codon of the downstream gene is shown.

The strategy used for the assembly of the constructs for cadmium biosensing and adsorption is summarized in Figure 1, and the DNA sequences of the expression regions of recombinant plasmids involved in the study are shown in Supplementary Figure 1. The genetic element coding for the transcriptor CadR and the bidirectional *cad* promoter region (NCBI Accession No. AF333961) was synthesized, and this fragment was then cloned as a *Bgl*II and *Xba*I fragment into pET-21a to generate pPcad. Firstly, a promoterless *mcherry* gene was amplified from pT-RFP, and subcloned into the *Nde*I and *Hin*dIII sites of pPcad to generate pPcad-RFP, which is designed as a single-signal output cadmium biosensor. Secondly, a DNA fragment containing the ribosome binding site (RBS) and the *egfp* gene was amplified from pT-GFP, and fused with the *mcherry* gene by an overlapping extension PCR as described previously (Maruthamuthu et al., 2015a) to generate pPcad-RFP-GFP, which is designed as a dual-signal output cadmium biosensor. Finally, a DNA fragment containing an extra RBS and the *lacZ*α gene was amplified from pPlac-lacZα, and fused with the *egfp* gene to construct the pPcad-RFP-GFP-lacZα plasmid, which is designed as a triple-signal output cadmium biosensor.

Besides some low molecular weight metal binding peptides (Maruthamuthu et al., 2015b, 2018), metal binding domains derived from MerR family members have been demonstrated to be efficiently displayed on the surface of bacterial cells for target metal ions removal (Tao et al., 2016; Hui et al., 2018b). In this study, cadmium binding domain (CdBD) derived from CadR was fused with the C-terminus of the surface display protein Lpp-OmpA to generate the chimera protein Lpp-OmpA-CdBD. The synthetic DNA fragments encoding Lpp-OmpA and Lpp-OmpA-CdBD were cloned into the pPcad using *Nde*I and *Hin*dIII to construct pPcad-LOA (control) and pPcad-CdBD, respectively. The latter is designed as a Cd(II) inducible cadmium adsorptive vector. Then, the DNA fragment coding for the RBS and the single reporter eGFP was fused with the Lpp-OmpA-CdBD cassette to generate pPcad-CdBD-GFP, which is designed as a Cd(II) inducible cadmium adsorptive and single-signal output biosensing vector. Likewise, the dual reporter eGFP-lacZα fusion cassette was fused into pPcad-CdBD by the overlapping extension PCR to generate pPcad-CdBD-GFP-lacZα, which is designed as a Cd(II) inducible cadmium adsorptive and dual-signal output biosensing vector.

**Figure 2:**
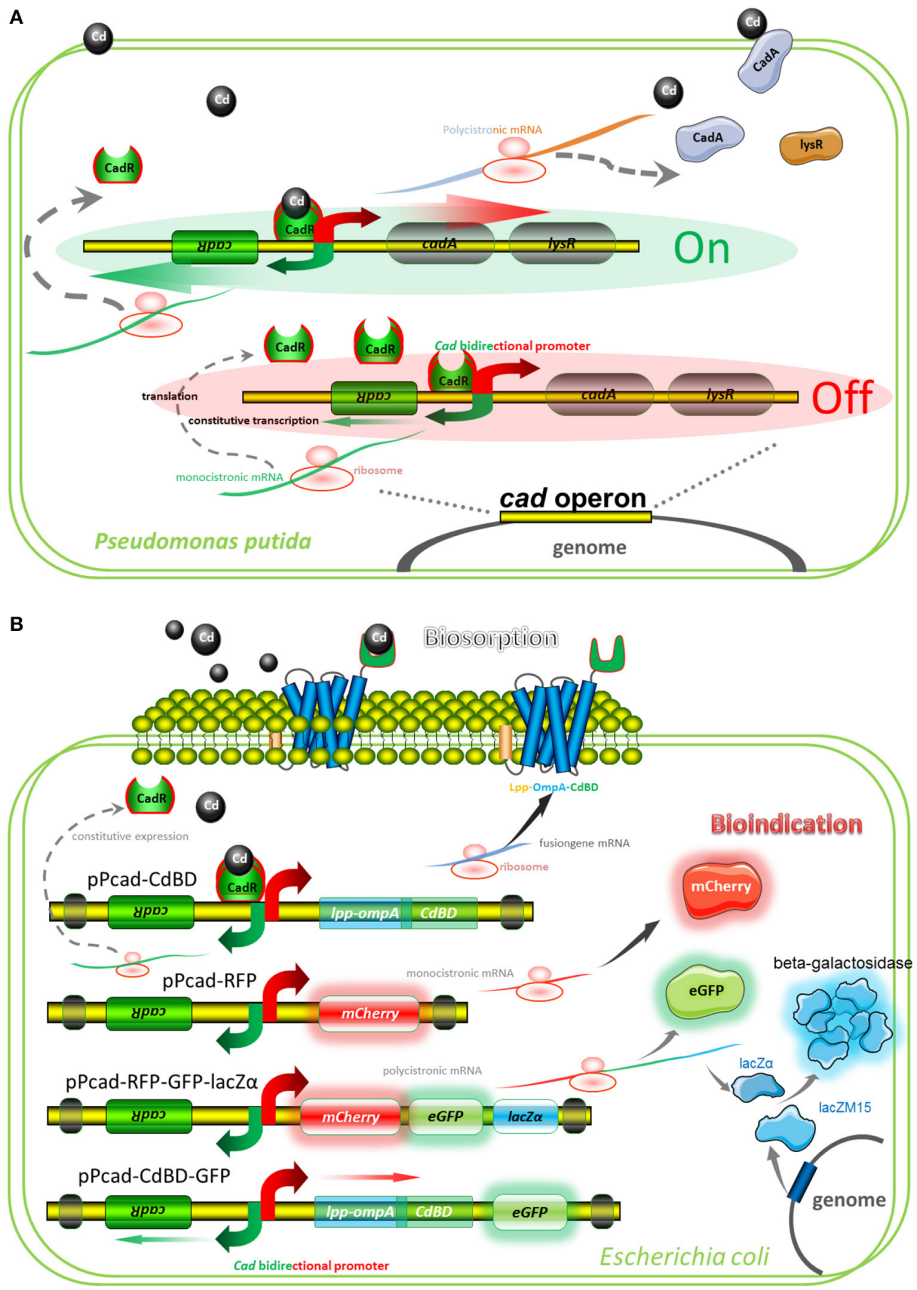
Models for natural and artificial *cad* operons. **(A)** Model for natural *cad* operon-encoded cadmium resistance of *Pseudomonas putida* 06909. The model involves the following proteins: CadR, which is related to the MerR family of response regulators; CadA, a cadmium-transporting ATPase; LysR, a LysR family response regulator, which may be involved in bacterium-fungus interactions. **(B)** Models for artificial *cad* operon-encoded bioindication and biosorption of Cd(II). The models involve the following proteins: Lpp-OmpA-CdBD, an outer membrane anchoring chimera protein for surface display of metal binding domain derived from CadR; mCherry and eGFP, fluorescent reporters; lacZα, an enzymatic reporter which is expressed under the control of *cad* promoter driven by Cd(II), and lacZM15, a protein that is synthesized under the control of host *lac* promoter driven by IPTG. After active tetrameric β-galactosidase is finally assembled, enzymatic activity is then determined.

A well-characterized cadmium resistance system is the *cad* operon in *Pseudomonas putida* (Lee et al., 2001; Brocklehurst et al., 2003). Natural *cad* operon is demonstrated to comprise of a regulatory gene plus an efflux-ATPase (Figure 2A). The sensing element is CadR, a dimer protein, which represses transcription of the *cad* operon in the absence of Cd(II) but activates transcription in the presence of Cd(II). Divalent cadmium ion, which has entered the cytoplasm, can finally be exported by the CadA Cd(II) efflux-ATPase. Although CadA and LysR belong to be a single transcriptional unit, LysR is not absolutely required for Cd(II) resistance (Lee et al., 2001). Engineered *cad* operons involved in our study are summarized in Figure 2B, and artificial genetic elements are divergently transcribed from *cadR*. The CadA-LysR cassette can be substituted by the Lpp-OmpA-CdBD cassette and the mCherry cassette for cadmium bioadsorption and fluorescent indication, respectively. More importantly, a single transcriptional unit assembled by multiple functional elements can substitute for the CadA-LysR cassette. It allows multiple genes to be co-transcribed into a polycistronic mRNA strand, followed by coupled expression of multiple reporters and surface-displayed CdBD for multiple signals output biosensing and bioadsorption of cadmium.

### Induction Assays

*E. coli* TOP10 was transformed with pPcad-RFP, pPcad-RFP-GFP, and pPcad-RFP-lacZα using a CaCl_2_-mediated transformation method (Hui et al., 2018c). For the sensitivity detections, single colonies from three transformation plates were used to inoculate 3 mL of M9 medium containing 50 μg/mL ampicillin in a 15-mL bio-reaction tube (Jet, Guangzhou, China), and cultured overnight in a 37^o^C shaking incubator at 200 rpm. One hundred microliters of overnight culture was inoculated into 10 mL fresh M9 medium in a 50-mL bio-reaction tube (Jet, Guangzhou, China) with three repetitions, and the cultures were grown at 37^o^C for 4 h with rotation at 200 rpm. The exponential phase cultures were combined, and then sub packed into 15-mL bio-reaction tubes. The first tube was filled with 4 mL of culture spiked with 5 μL of 10 mM Cd(II) (the final concentration of Cd(II) is 12.5 μM), and the other tubes were filled with 2 mL of culture. A double dilution method was used to obtain 6.25, 3.125, 1.56, 0.78, 0.39, 0.2, and 0.1 μM Cd(II) exposure groups. The resultant tubes were cultured at 30^o^C for 20 h before assessment of reporter signals.

For the specificity detections, three milliliters of exponential cultures of three recombinant *E. coli* biosensors were spiked with 15 μL of 1 mM various metal ions, followed by culturing at 30^o^C for 20 h.

For the comparison of responses toward Cd(II) and Hg(II), three milliliters of exponential cultures of three recombinant *E. coli* biosensors were exposed to 0, 0.625, 1.25, 2.5, 5, 10, and 20 μM Cd(II) or Hg(II) by using a double dilution method as described above. The cultures were then grown at 30^o^C for 20 h before assessment of reporter signals.

For the detection and adsorption of Cd(II), four different recombinant *E. coli* TOP10 harboring artificial *cad* operons pPcad-LOA, pPcad-CdBD, pPcad-CdBD-GFP, and pPcad-CdBD-GFP-lacZα were cultured in supplemented M9 medium at 37^o^C for 4 h with rotation at 200 rpm, then exposed to 0, 0.1, 0.2, 0.39, 0.78, 1.56, 3.125, 6.25, and 12.5 μM CdCl_2_ by using a double dilution method as described above. The resultant cultures were grown at 30^o^C for 20 h before measurement of reporter signals and cadmium binding capacities.

### Measurements of Reporter Signals

The fluorescent signals generated from the induced *E. coli* biosensors were measured with a Lumina fluorescence spectrometer (Thermo, USA) as previously described (Hui et al., 2018c). The excitation wavelength was set at 587 nm and the intensity of emitted fluorescence of mCherry at 610 nm was recorded. Subsequently, the excitation wavelength was set at 488 nm and the intensity of emitted fluorescence of eGFP at 507 nm was recorded. The fluorescence intensities were normalized by dividing the fluorescence intensity by the OD_600_ value of the same sample. The lacZα production-inducing β-galactosidase activity of induced culture (Guo et al., 2019) was measured as described by Miller with ONPG as the substrate (Miller, 1972).

### Measurement of Cells-Associated Cd(II)

The induced *E. coli* cells were harvested from M9 medium by centrifugation at 3,500 rpm for 10 min, washed extensively with ddH_2_O, dried (80°C, 12 h), weighed, and digested. The metal ion content in the samples was measured using an atomic absorption spectrometer (PerkinElmer, USA) as described previously (Hui et al., 2018a).

### Fluorescence Imaging Based on Cd(II)-Charged Beads Binding Assay

Strong cation exchanger SP sepharose fast flow was washed with distilled water extensively, and treated with two volumes of 100 mM CdCl_2_ in distilled water. The sepharose beads were then washed extensively with ddH_2_O to remove any excess of Cd(II), and suspended in one volume of ddH_2_O. Induced TOP10/pPcad-CdBD-GFP cells were harvested, washed three times with ddH_2_O, and suspended with 10 volumes of ddH_2_O. The sepharose beads precharged with Cd(II) were then mixed with cell suspensions gently. The mixture was mounted onto a clear glass slide, and then visualized using a Nikon Eclipse Ni fluorescence microscope (Tokyo, Japan) as described previously (Hui et al., 2019). A FITC filter (excitation at 475–490 nm and emission at 505–535 nm) was used to detect eGFP fluorescence, and representative images were captured. After fluorescent observation of cell adhesion, the suspension above was further mixed with an equal volume of 100 mM Cd(II) solution and stirred gently for 5 min. The mixture was mounted onto a clear glass slide, and another fluorescent observation was then performed.

## Results and Discussion

### Cadmium Detection With Three Different Cadmium Biosensors

Single-signal output cadmium biosensor TOP10/pPcad-RFP, dual-signal output cadmium biosensor TOP10/pPcad-RFP-GFP, and triple-signal output cadmium biosensor TOP10/pPcad-RFP-GFP-lacZα were firstly exposed to a range of Cd(II) to assess their tolerance, detection range and reporter responses. As shown in Supplementary Figure 2, three biosensor cells could tolerate 25 μM Cd(II), and the growth of recombinant strains was decreased slightly with 50 μM Cd(II) exposure. In addition, previous studies have demonstrated that there was no significant difference in the growth curves of TOP10 with 0–1.0 mM IPTG exposure, and β-galactosidase activity derived from recombinant TOP10 harboring lacZα peptide expression vectors was not elevated above 0.1 mM IPTG induction (Guo et al., 2019; Hui et al., 2020b). Finally, three different biosensors were demonstrated to detect values as low as 0.1 μM Cd(II). Upon exposure to 0.1–3.125 μM Cd(II), reporter signals of three different biosensors all increased. The maximum reporter responses were obtained at 3.125 μM and above, but decreased at Cd(II) concentration higher than 50 μM, due to bacterial growth inhibitory (data not shown).

**Figure 3:**
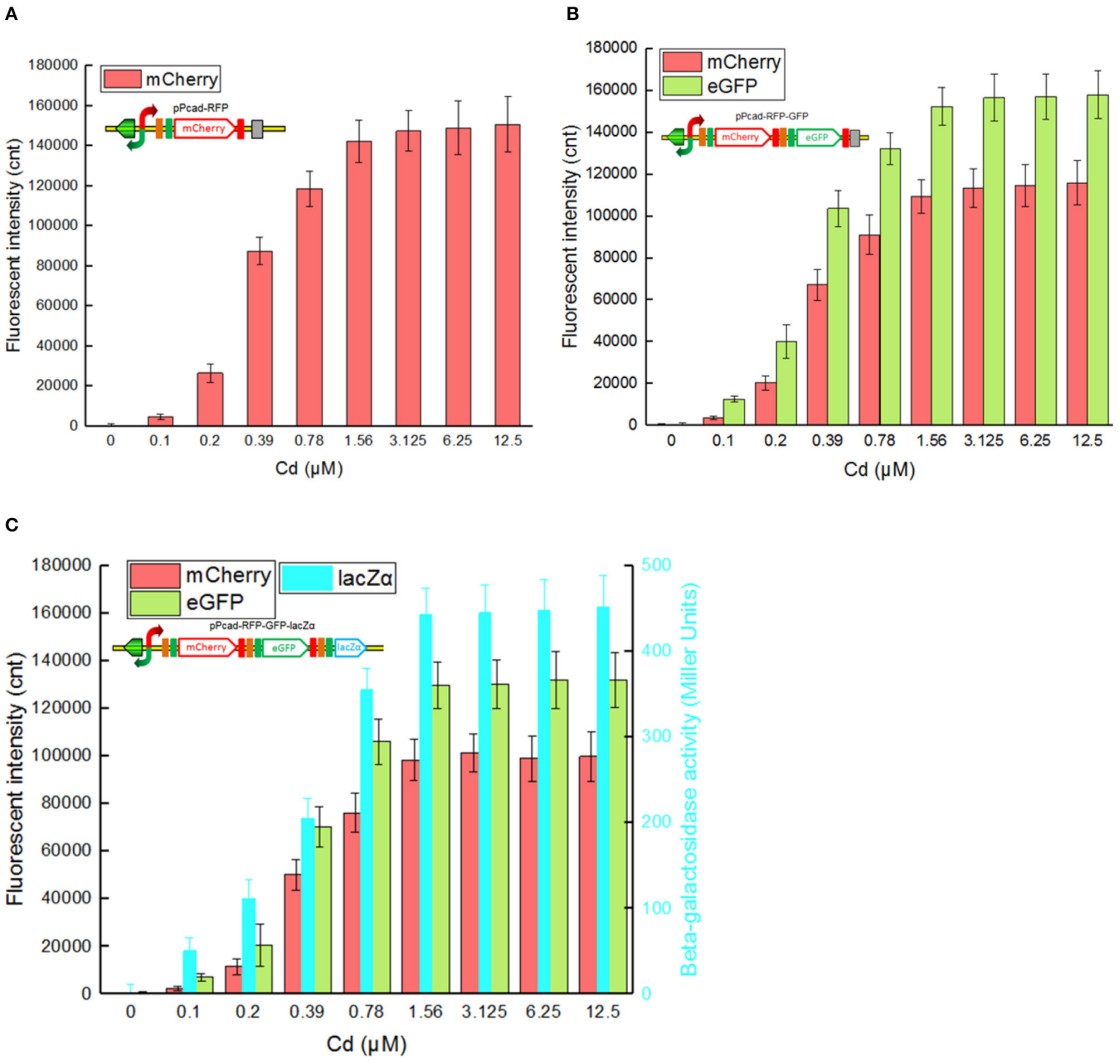
Three cadmium biosensors were exposed to gradient concentrations of Cd(II). After induction with 0–12.5 μM Cd(II) at 30^o^C for 20 h, Fluorescent signals (mCherry and eGFP) and enzymatic signal (lacZα) were all determined. **(A)** TOP10/pPcad-RFP. **(B)** TOP10/pPcad-RFP. **(C)** TOP10/pPcad-RFP-lacZα. Fluorescence intensity and enzymatic activity values were normalized using the absorbance at 600 nm. The data were obtained by subtracting the value of recombinant *E. coli* with 0 μM Cd(II) exposure from that of each group. Data are representative of three independent experiments, and values are expressed as mean ± *SD*.

The greatest disadvantage of coupled translation in the artificial operons is the decreased expression of the downstream gene, and this is related to the distance and DNA sequence between the termination of the upstream gene and the start of the downstream one (Levin-Karp et al., 2013). To minimize the report level decline of the downstream reporter, the stop codon of the upstream reporter is designed to be overlapped with the RBS of the downstream reporter gene in this study (Figure 1) as described previously (Maruthamuthu et al., 2015a; Hui et al., 2018c). On the other hand, three reporter genes are assembled into the polycistronic *cad* operons based on their response abilities. The eGFP reporter with high emitted fluorescent intensity is inserted behind the mCherry reporter with low emitted fluorescent intensity, and the highest sensitive reporter lacZα among them is located behind eGFP. As a result, the mCherry fluorescent signal decreased about 20% in the dual-signal output biosensor TOP10/pPcad-RFP-GFP when compared with the single-signal output biosensor TOP10/pPcad-RFP, and further decreased about 10% in the triple-signal output biosensor TOP10/pPcad-RFP-GFP-lacZα (Figure 3). Compared with the eGFP fluorescent signal in TOP10/pPcad-RFP-GFP, that in TOP10/pPcad-RFP-GFP-lacZα decreased about 15% (Figure 3). All three biosensors can detect Cd(II) at concentrations as low as 0.1 μM Cd, and it is similar with the single GFP output biosensor based on artificial *cadCA* operon (0.082 μM) (Kumar et al., 2017) and artificial *cad* operon (0.09 μM) (Bereza-Malcolm et al., 2017). More importantly, the response strength of three reporters is lacZα > eGFP > mCherry in the triple-signal output biosensor (Figure 3C). The above results have demonstrated that the designed multiple-signal output biosensor is instrumental in Cd(II) biosensing.

**Figure 4:**
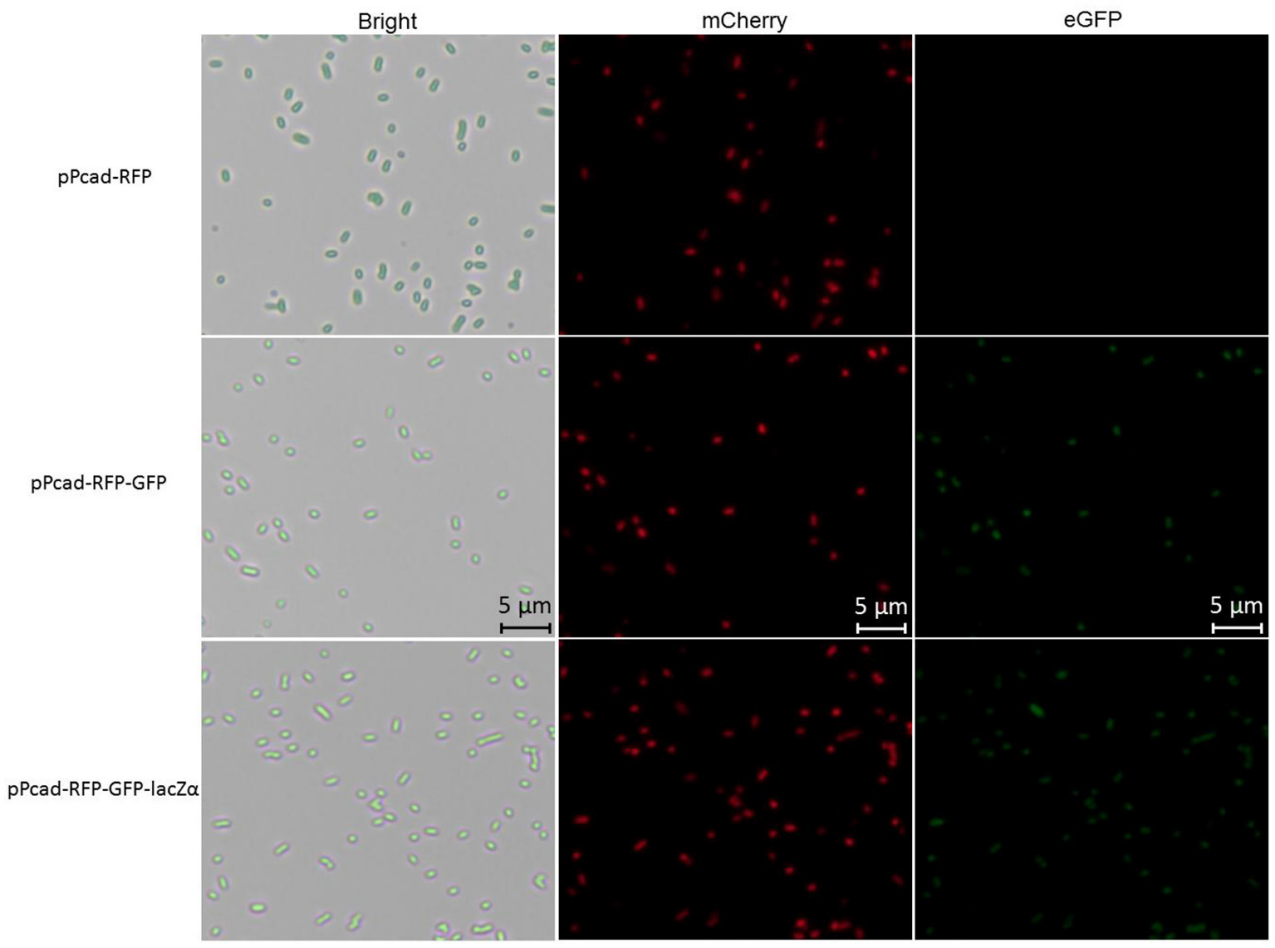
Fluorescence images of TOP10/pPcad-RFP, TOP10/pPcad-RFP-GFP, and TOP10/pPcad-RFP-GFP-lacZα exposed to 5 μM Cd(II) at 30^o^C for 20 h. *E. coli* cells were visualized using a fluorescence microscope (×400 magnification). The fluorescence imaging of mCherry upon excitation at 545–565 nm and emission detection between 580 and 620 nm, and fluorescence imaging of eGFP upon excitation at 475–490 nm and emission detection between 505 and 535 nm.

Compared with traditional single-fluorescence output biosensor TOP10/pPcad-RFP, dual-fluorescence output biosensor TOP10/pPcad-RFP-GFP and TOP10/pPcad-RFP-GFP-lacZα provide another choice for detection, and will be useful when the overlapped background fluorescence of the sample exists. The fluorescent images of TOP10/pPcad-RFP, TOP10/pPcad-RFP-GFP, and TOP10/pPcad-RFP-GFP-lacZα with 5 μM Cd(II) exposure were shown in Figure 4. Cd(II) exposure successfully induced single- and dual-colored fluorescent signals from three kinds of Cd(II) whole-cell biosensors. No fluorescent signals were detected from three biosensors without Cd(II) exposure (Data not shown).

### Metal Selectivity Detection With Three Cadmium Biosensors

**Figure 5:**
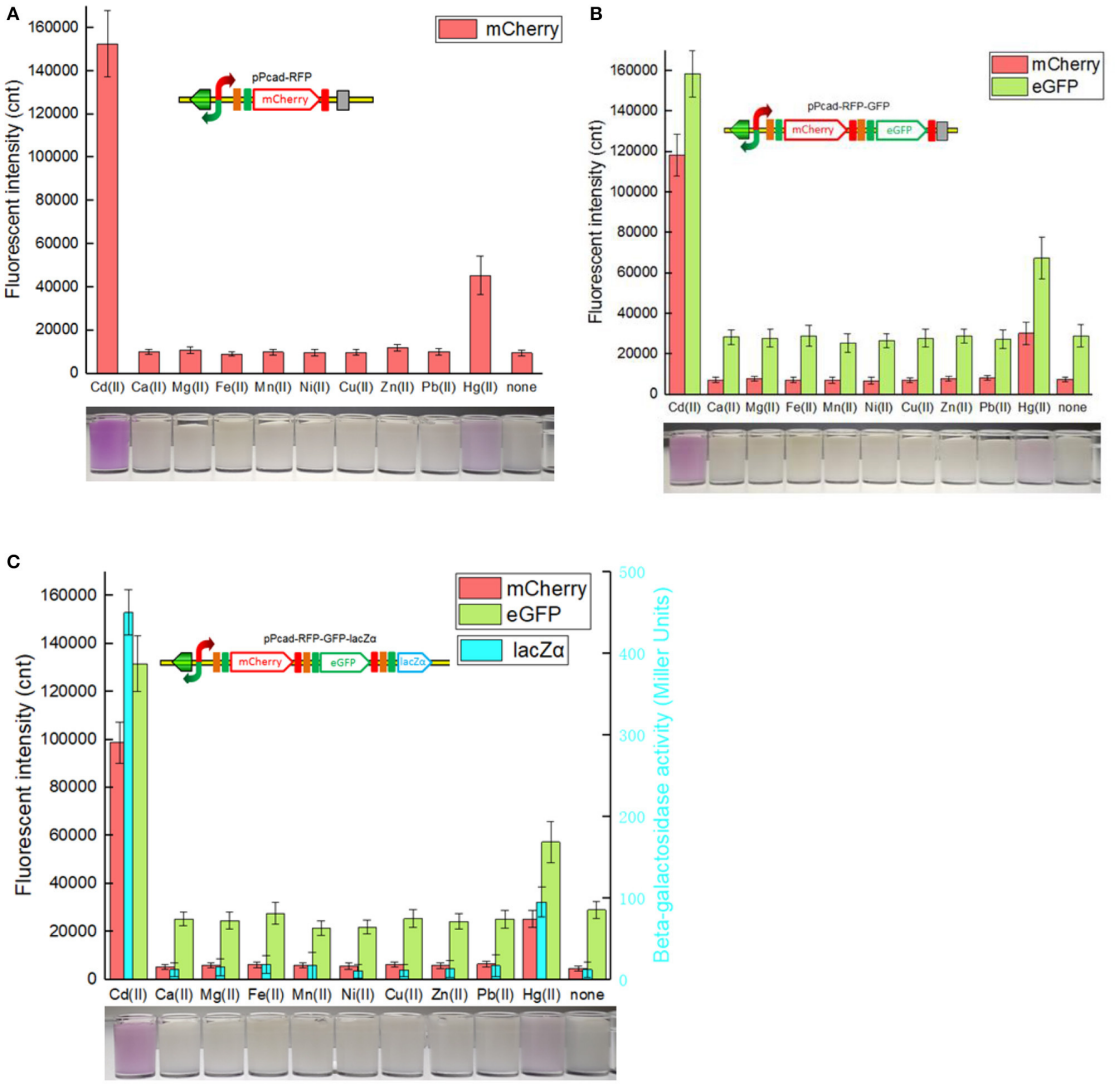
Three cadmium biosensors were exposed to a range of metal ions at 5 μM. After induction with 5 μM various metal ions at 30^o^C for 20 h, three kinds of reporter signals were determined. The bottom panels are the typical photographs of induced *E. coli* biosensors with 5 μM corresponding metal ions. **(A)** TOP10/pPcad-RFP. **(B)** TOP10/pPcad-RFP-GFP. **(C)** TOP10/pPcad-RFP-GFP-lacZα. Fluorescence intensity and enzymatic activity values were normalized using the absorbance at 600 nm. Data are representative of three independent experiments, and values are expressed as mean ± *SD*.

As expected, the specificity assay found that three cadmium biosensors were all responsive to Cd(II). In addition, very strong signal outputs were obtained in the presence of 5 μM Hg(II). The accumulation of mCherry responsive to Cd(II) and Hg(II) were enough to be visible to the naked eye in all three biosensors (Figure 5). The results are consistent with previous studies that *cad* operon derived from *Pseudomonas putida* 06909 responds to Cd(II) > Hg(II), and not to the other divalent cations (Lee et al., 2001; Tao et al., 2013; Cayron et al., 2019). Natural *cadR* along with its divergent promoter were used as the sensing element in the study, and it resulted in the response to Hg(II). The key amino acid residues of MerR-like regulatory protein were demonstrated to be responsible for its metal specificity (Hakkila et al., 2011; Cayron et al., 2019). The preferred Cd-specific CadR mutants may be generated by site-directed mutagenesis, and further to assemble multiple-signal output biosensors with high selectivity toward Cd(II).

### Different Responses of Three Cadmium Biosensors Toward Cd(II) and Hg(II)

**Figure 6:**
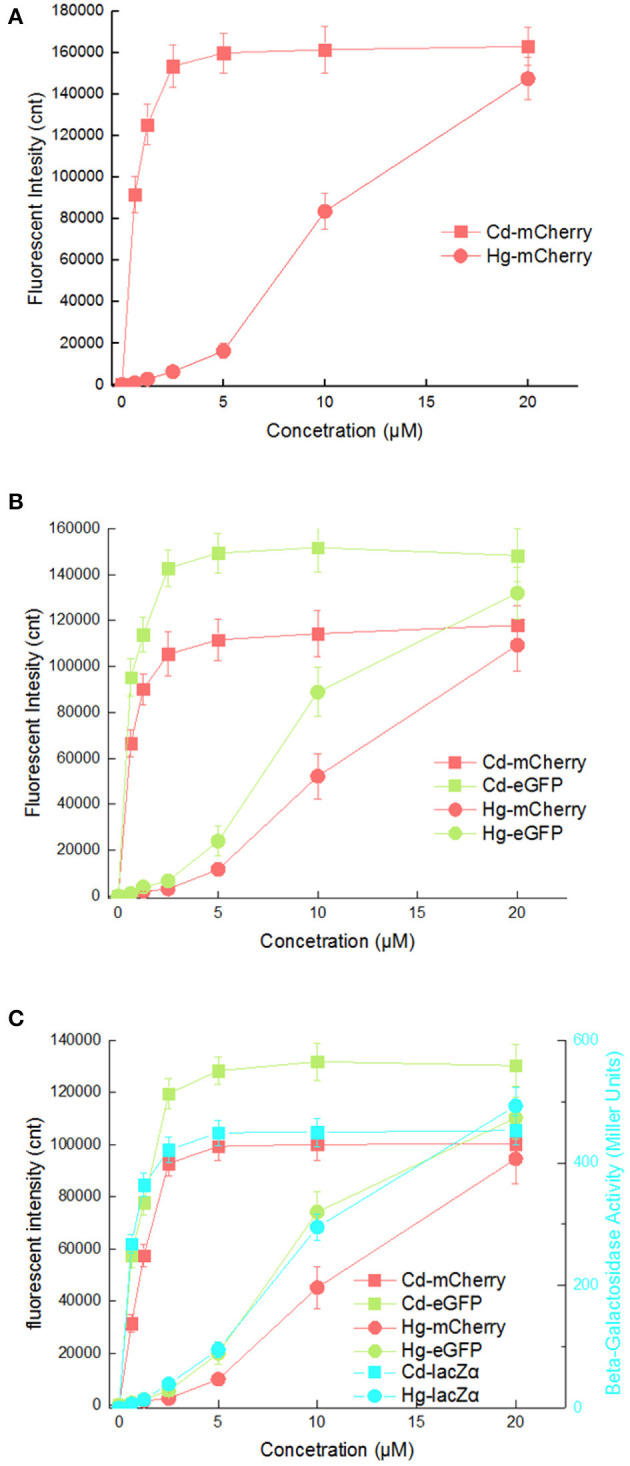
Comparison of reporter signal changes generated by three cadmium biosensors after exposure to different concentrations of Cd(II) and Hg(II). After induction with various concentrations of Cd(II) and Hg(II) at 30^o^C for 20 h, three kinds of reporter signals were determined. **(A)** TOP10/pPcad-RFP. **(B)** TOP10/pPcad-RFP-GFP. **(C)** TOP10/pPcad-RFP-GFP-lacZα. Fluorescence intensity and enzymatic activity values were normalized using the absorbance at 600 nm. The data were obtained by subtracting the value of recombinant *E. coli* with no metal exposure from that of each group. Data are representative of three independent experiments, and values are expressed as mean ± *SD*.

It is possibly disadvantageous for a biosensor to respond to two kinds of heavy metal ions, as this would limit its applications. The response curves of three cadmium biosensors toward Cd(II) and Hg(II) were then compared. As shown in Figure 6, all three biosensors responded well to 0.625 μM Cd(II) by increasing rapidly, and maximum responses were achieved at above 2.5 μM Cd(II). However, obvious responses to Hg(II) from all three biosensors were observed at above 2.5 μM Hg(II) under the same culture condition, and increased within the range 2.5–20 μM Hg(II). The suitable detection ranges of Cd(II) (0–2.5 μM) and Hg(II) (2.5μM above) do not overlap in any of the three biosensors.

Mercury, as a transition metal, is similar in structure to cadmium. It is not surprising that artificial *cad* operons respond to Hg(II) (Tauriainen et al., 1998; Bereza-Malcolm et al., 2017). However, the response characteristic of artificial *cad* operon toward Hg(II) has not been carefully evaluated. Inorganic mercury compounds including HgCl_2_ are dispersed in an aqueous phase in molecular form rather than in ionic form, so the concentration of extractable Hg(II) that can be sensed by whole-cell biosensors is actually very low. It is possible the main reason for the sensitivity of these existing whole-cell Hg(II) biosensors does not satisfy the requirements for application (Hansen and Sorensen, 2000; Jouanneau et al., 2011). The *mer* operon is the only natural heavy metal resistance system that has been successfully redesigned to become the Hg(II) biosensors with luciferase as a single reporter (Hansen and Sorensen, 2000; Rasmussen et al., 2000; Du et al., 2019). However, the response of artificial *cad* operons to Hg(II) suggests the potential of this strategy for the development of extractable Hg(II) biosensors. To simultaneously detect and distinguish both bioavailable Cd(II) and Hg(II), an integrated whole-cell biosensor is expected to be developed by employing the dual-sensing *cad* operon and single-sensing *mer* operon as separate metal-sensing elements, and different reporters as signal output elements.

### Integrated Cadmium Biosensing and Adsorption Based on Artificial *Cad* Operons

**Figure 7:**
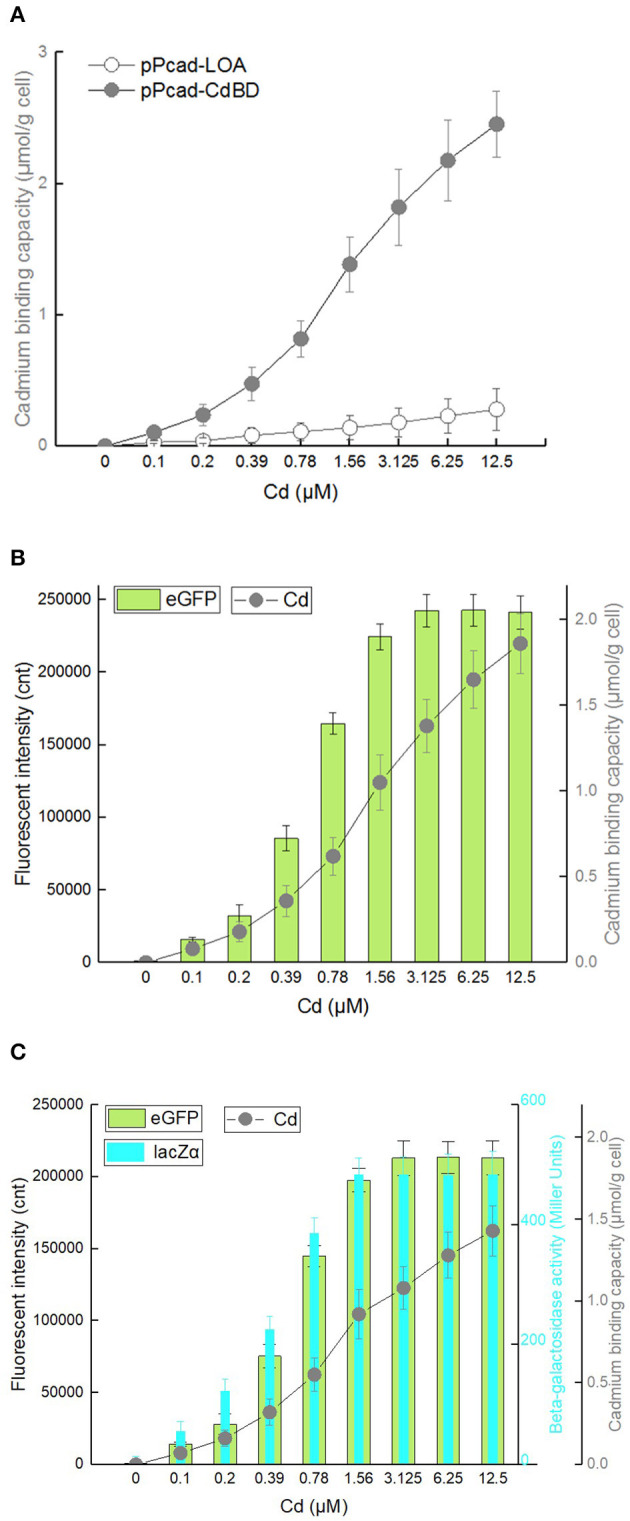
Cadmium adsorption and biosensing by recombinant *E. coli* harboring three artificial *cad* operons. After exposure to 0–12.5 μM Cd(II) at 30^o^C for 20 h, cadmium binding capacity, eGFP fluorescence intensity and β-galactosidase activity were determined separately. **(A)** TOP10/pPcad-CdBD and TOP10/pPcad-LOA (as control). **(B)** TOP10/pPcad-CdBD-GFP. **(C)** TOP10/pPcad-CdBD-GFP-lacZα. Fluorescence intensity and enzymatic activity values were normalized using the absorbance at 600 nm. The data were obtained by subtracting the value of recombinant *E. coli* with 0 μM Cd(II) exposure from that of each group. Data are mean ± *SD* from three independent assays each from three independent cultivations.

To investigate whether the surface-displayed CdBD retains the ability to capture Cd(II), TOP10/pPcad-LOA, and TOP10/pPcad-CdBD were first induced with increasing concentrations of Cd(II). The heavy metal binding capacity was previously demonstrated to be positively correlated with both the amount of displayed metal binding protein and the concentration of incubated target metal ions (Wei et al., 2014; Hui et al., 2018b). As expected, compared with the control group TOP10/pPcad-LOA, Cd(II) binding capacity of TOP10/pPcad-CdBD was significantly enhanced with increasing concentrations of incubated Cd(II). As shown in Figure 7A, TOP10/pPcad-CdBD with the surface-displayed CdBD was able to capture Cd(II) with a capacity of about 2.46 μmol/g cell at 12.5 μM Cd(II) exposure level, which is 8.77-fold higher than that of the control group without CdBD display.

Biodetection and bioremediation of toxic heavy metal including cadmium, as an important issue in environmental protection and governance, are usually achieved using a single-signal output biosensor (Kim et al., 2016; Bereza-Malcolm et al., 2017; Kumar et al., 2017) and surface display of Cd(II) binding protein (Sousa et al., 1996; Tafakori et al., 2012; Tao et al., 2016; Tang et al., 2018), respectively. A dual functional lead sensing and removal system was previously developed by co-expression of GFP and OmpC-lead binding peptide under the control of *zraP* promoter (Maruthamuthu et al., 2015a). A similar strategy was successfully used to construct a toxic bisphenol-A sensing and adsorption system (Maruthamuthu et al., 2017). Integration of cadmium biosensing and adsorption in a single genetic construct which simulates natural *cad* operon was developed in this study for the first time. To maximize the Cd(II) binding capacity, the CdBD display genetic element was located at the upstream of sensing elements for enhanced display efficiency. Single-signal output sensing-adsorptive TOP10/pPcad-CdBD-GFP, and dual-signal output sensing-adsorptive TOP10/pPcad-CdBD-GFP-lacZα were able to adsorb Cd(II) with a capacity of about 1.86 μmol/g cell and 1.43 μmol/g cell at 12.5 μM Cd(II) exposure level, which are 6.64- and 5.11-fold higher than that of the control group (Figures 7B,C), respectively. The metal-binding capability of peptides was usually correlated with their expression level and display efficiency (Hui et al., 2018b, 2019). However, metal adsorption capacities could be further enhanced with increased concentration of incubated target metal even when the surface display level of peptides was constant (Wei et al., 2014; Maruthamuthu et al., 2015b, 2018; Tao et al., 2016; Hui et al., 2018a). Similar to the previous studies, we found that the responses of two sensing-adsorptive constructs toward Cd(II) were not increased at 3.125 μM above, but significant higher cadmium binding capacities were still observed when the concentration of Cd(II) exposure was further increased (Figures 7B,C).

The response changes from the downstream reporters in Cd(II) sensing-adsorptive constructs (Figures 7B,C) were similar with that in Cd(II) biosensors described above (Figures 3B,C). Importantly, the maximum report level of TOP10/pPcad-CdBD-GFP (eGFP, 242441 cnt) and TOP10/pPcad-CdBD-GFP-lacZα (eGFP, 213200; lacZα, 483 MU) were achieved at 3.125 μM Cd(II) above, and significantly higher than that of TOP10/pPcad-RFP-GFP (eGFP, 156503 cnt) and TOP10/pPcad-RFP-GFP-lacZα (eGFP, 130077; lacZα, 445 MU), respectively. There are two reasons that probably explain this phenomenon. Firstly, the molecular weight of Lpp-OmpA-CdBD (228 aa, 2.48 kDa) is lower than the molecular weight of mCherry (236 aa, 2.88 kDa). It is well-understood that the expression of low molecular weight proteins is usually energy-saving for host cells (Mahalik et al., 2014), and is beneficial for the enhanced production of the downstream reporters (Hui et al., 2018c, 2020b). Secondly, it is unclear whether enriched Cd(II) on the surface of sensing-adsorptive engineered cells contributes to the elevated concentration of Cd(II) *in vivo*, which can eventually induce stronger reporter expression. In summary, our results demonstrate that the designed multiple-signal output sensing and adsorptive construct is instrumental in simultaneous detection and removal of bioavailable Cd(II).

Extensive studies have demonstrated that whole-cell biosensors could detect target molecules in polluted water (Yin et al., 2016; Maruthamuthu et al., 2017; Cui et al., 2018; Hui et al., 2020a), soil extract (Rasmussen et al., 2000; Yoon et al., 2016a,b)(Rasmussen et al., 2000; Yoon et al., 2016a,b), and milk (Kumar et al., 2017) by introducing these liquid samples to be tested into the culture system. Biotoxic molecules could be efficiently removed by whole-cell bioadsorbers from polluted water and soils (Wei et al., 2014; Maruthamuthu et al., 2017; Tang et al., 2018). The dual function engineered bacteria developed in this study can simultaneously sense and remove bioavailable Cd(II) from the surroundings, which would be more available in the polluted environment.

**Figure 8:**
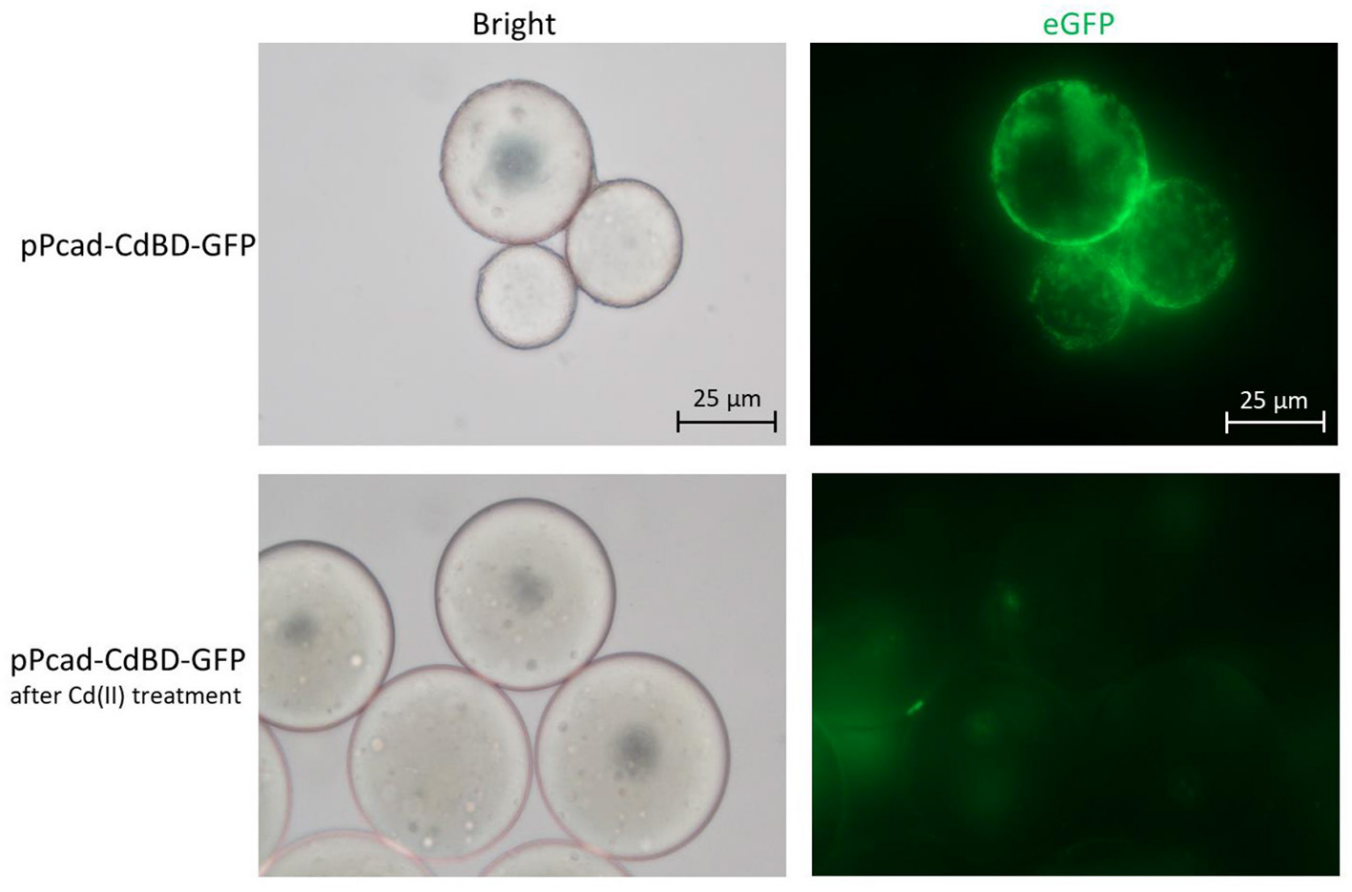
Adhesion of TOP10/pPcad-CdBD-GFP to cation exchange gel charged with Cd(II). After SP sepharose fast flow beads were precharged with Cd(II), recombinant bacterial cells were suspended with ddH_2_O, and mixed with the beads for the first fluorescent observation (up panel, ×400 magnification). After the addition of an equal volume of 100 mM Cd(II), the second fluorescent picture was captured (down panel, ×400 magnification).

The metal adsorption process was previously studied by either fluorescent staining of dead cells (Sousa et al., 1996; Xu and Lee, 1999) or transmission electron micrograph of dead cells (Wei et al., 2014; Yin et al., 2016). However, multiple functional biosensing-adsorptive constructs facilitate the fluorescent observation of metal ion adsorption by live engineered cells. The eGFP fluorescence output can be directly used to monitor sensing-adsorptive engineered TOP10/pPcad-CdBD-GFP adhesion to beads coated with Cd(II) in real time. As shown in Figure 8, the large circles are SP sepharose beads which do not emit any fluorescence by themselves. The beads were precharged with Cd(II) to form an accessible Cd(II) affinity surface, and then the binding of induced TOP10/pPcad-CdBD-GFP made the beads light up in fluorescent green. Engineered cells could also be dissociated from the beads with excess Cd(II) washing, which demonstrates that the adsorption is specific to cadmium. These results present a proof of concept that sensing-adsorptive engineered cells can be used for live observation in real time, which facilitates the study of the mechanism and dynamics of the bioadsorption process.

## Conclusion

In this study, whole-cell multiple-signal biodetection and adsorption of cadmium were obtained using the assembly of artificial translationally coupled *cad* operons. The incorporation of multiple-signal output allowed measurement of the three designed Cd(II) biosensors through red fluorescence, green fluorescence, and β-galactosidase activity. Although the detection sensitivity was slightly decreased with the combination of multiple reporters, this method is able to provide more available detection ways. Versatile Cd(II) sensing-adsorptive engineered cells, constructed for the first time, were demonstrated to be instrumental in simultaneous detection and capture of bioavailable Cd(II), and will find plenty of use in the live observation for studying the bioadsorption process. Our findings show that it is worthwhile to develop multiple functional engineered cells for multiple-signal output biosensing and adsorption toward the target metal.

## Data Availability Statement

The raw data supporting the conclusions of this article will be made available by the authors, without undue reservation.

## Author Contributions

C-yH designed the experimental protocol and drafted the manuscript. YG, N-xZ, LL, HL, and H-jZ carried out the majority of the study. All authors read and approved the final manuscript.

## Conflict of Interest

The authors declare that the research was conducted in the absence of any commercial or financial relationships that could be construed as a potential conflict of interest.
