# Supplementary material for: Development of Cadmium Multiple-Signal Biosensing and Bioadsorption Systems Based on Artificial Cad Operons
Source: Front Bioeng Biotechnol. 2021 Feb 10;9:585617. doi: 10.3389/fbioe.2021.585617 (PMC7902519; doi:10.3389/fbioe.2021.585617)
Supplement: Supplementary file 1 [file Table_1.DOCX]

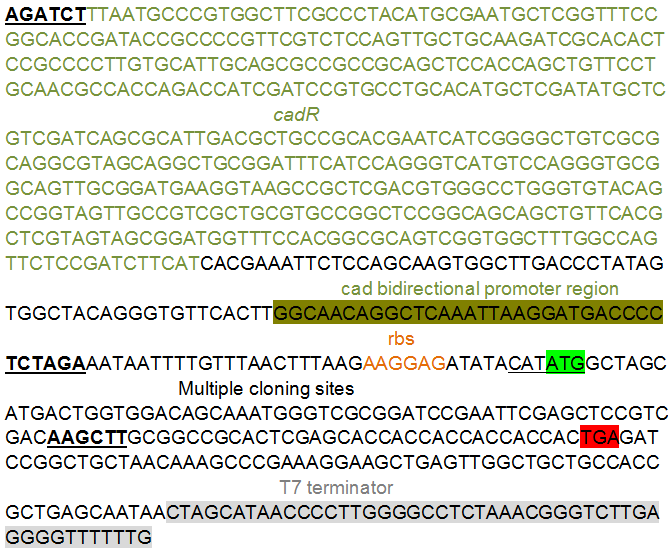


**pPcad**


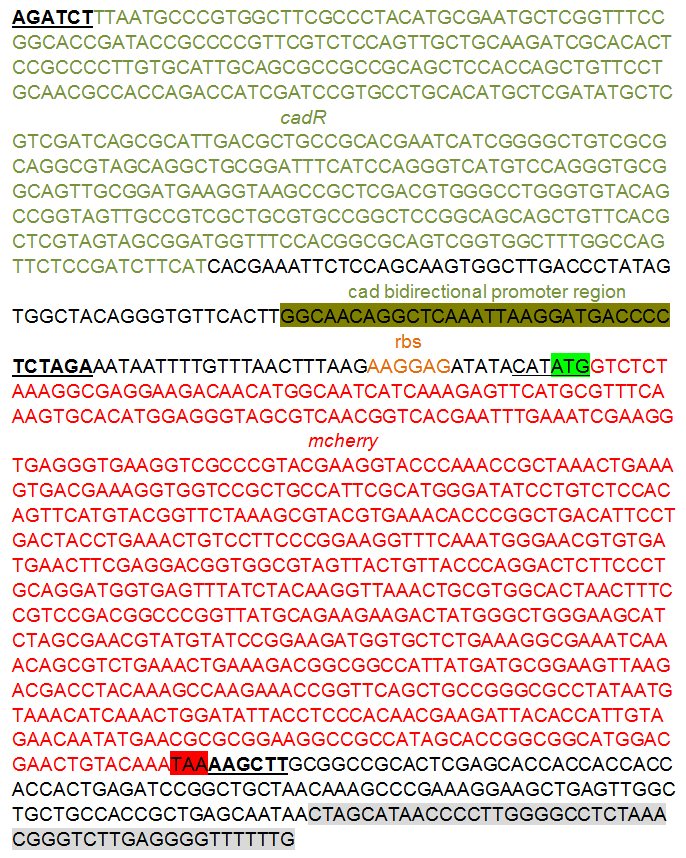


**pPcad-RFP**


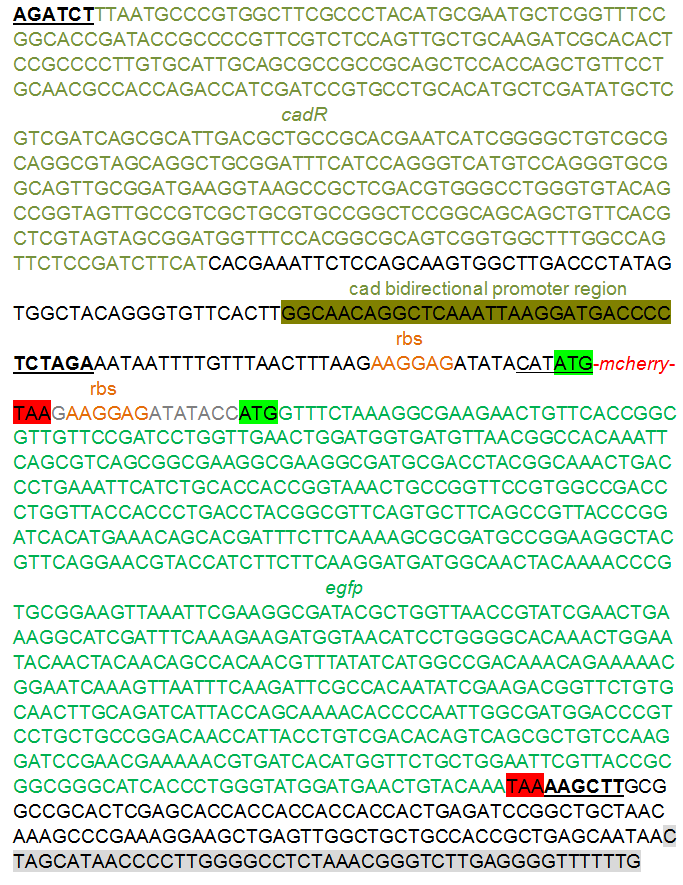


**pPcad-RFP-GFP**


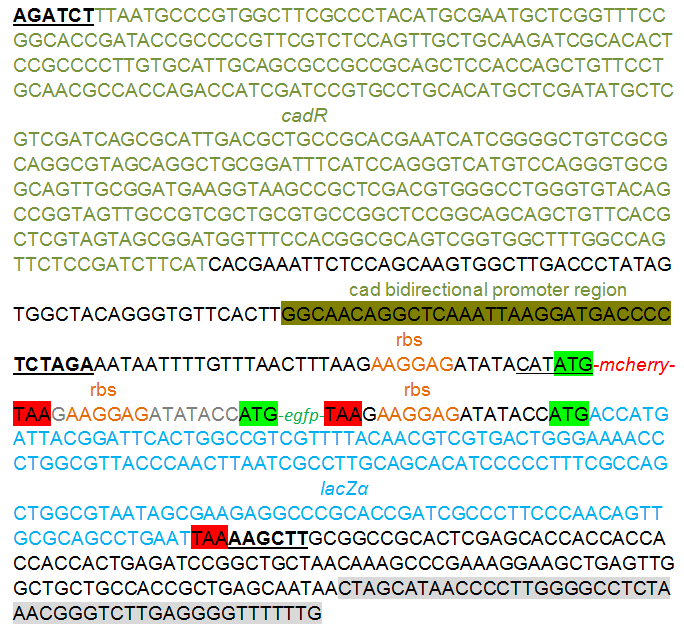


**pPcad-RFP-GFP-lacZα**


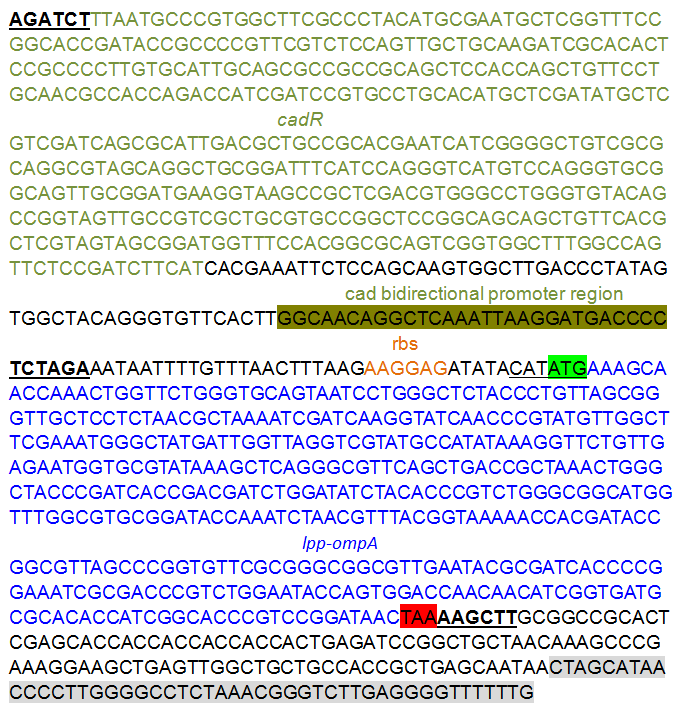


**pPcad-LOA**


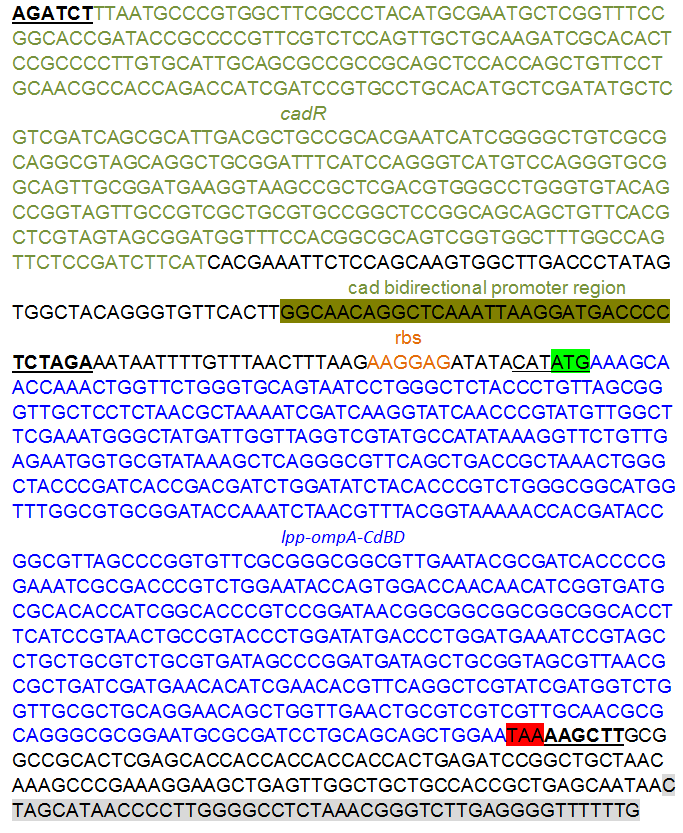


**pPcad-CdBD**


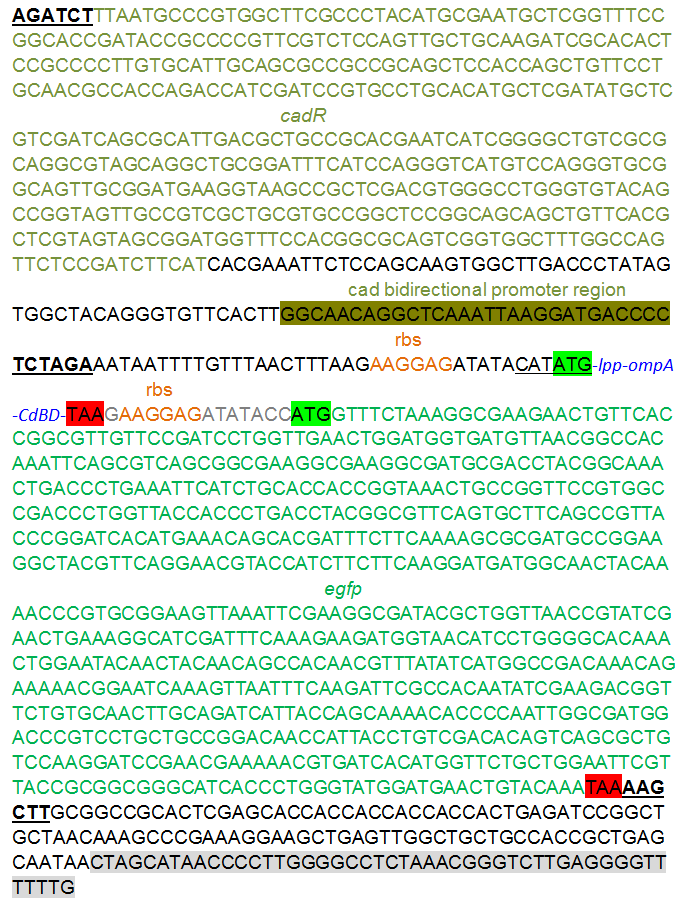


**pPcad-CdBD-GFP**


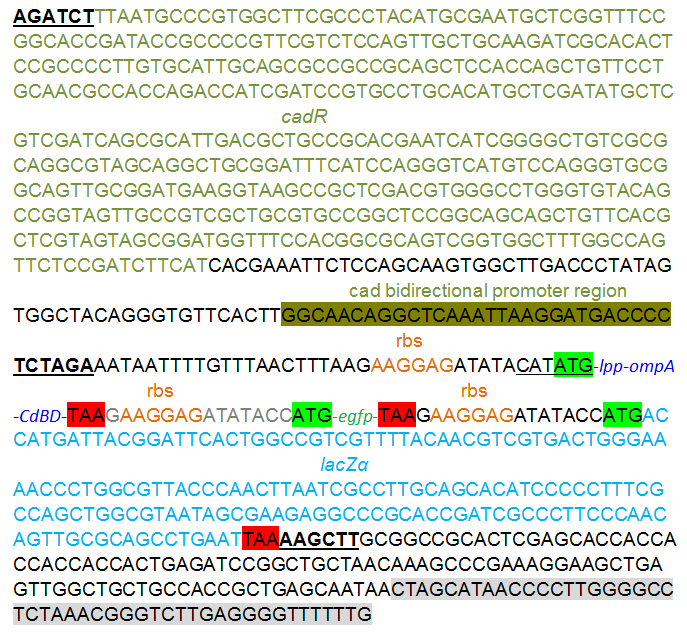


**pPcad-CdBd-GFP-lacZα**

**Fig. S1** The cloning/expression region of recombinant plasmids used in this study. DNA sequence and annotation data are all marked.


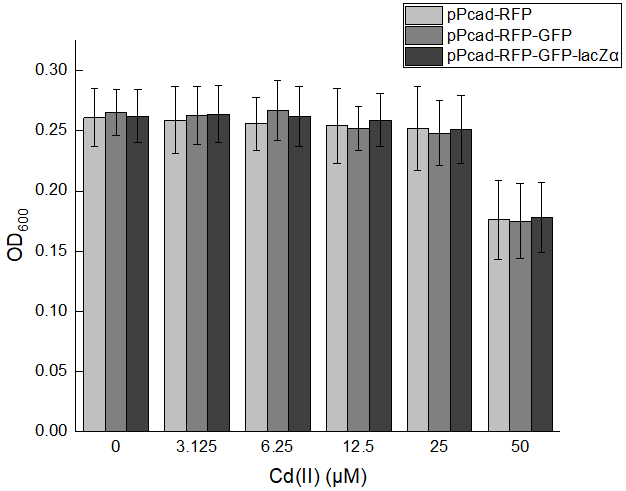


**Fig. S2** Toxic effects of Cd(II) on the growth of three biosensor cells.

Exponential cultures of Top10/pPcad-RFP, Top10/pPcad-RFP-GFP, and Top10/pPcad-RFP-GFP-lacZα were exposed to 0, 3.125, 6.25, 12.5, 25, 50 μM Cd(II), followed by culturing at 30 ^o^C for 20 h. The absorbance of each culture was determined at 600 nm.
